# Creation of an anthropomorphic CT head phantom for verification of image segmentation

**DOI:** 10.1002/mp.14127

**Published:** 2020-03-31

**Authors:** Robin B. Holmes, Ian S. Negus, Sophie J. Wiltshire, Gareth C. Thorne, Peter Young, The Alzheimer’s Disease Neuroimaging Initiative

**Affiliations:** ^1^ Department of Medical Physics and Bioengineering University Hospitals Bristol NHS Foundation Trust Bristol BS28HW UK; ^2^ Umea Functional Brain Imaging Center Umea University 901 87 Umea Sweden

**Keywords:** 3D printing, brain, image segmentation, imaging, phantom

## Abstract

**Purpose:**

Many methods are available to segment structural magnetic resonance (MR) images of the brain into different tissue types. These have generally been developed for research purposes but there is some clinical use in the diagnosis of neurodegenerative diseases such as dementia. The potential exists for computed tomography (CT) segmentation to be used in place of MRI segmentation, but this will require a method to verify the accuracy of CT processing, particularly if algorithms developed for MR are used, as MR has notably greater tissue contrast.

**Methods:**

To investigate these issues we have created a three‐dimensional (3D) printed brain with realistic Hounsfield unit (HU) values based on tissue maps segmented directly from an individual T1 MRI scan of a normal subject. Several T1 MRI scans of normal subjects from the ADNI database were segmented using SPM12 and used to create stereolithography files of different tissues for 3D printing. The attenuation properties of several material blends were investigated, and three suitable formulations were used to print an object expected to have realistic geometry and attenuation properties. A skull was simulated by coating the object with plaster of Paris impregnated bandages. Using two CT scanners, the realism of the phantom was assessed by the measurement of HU values, SPM12 segmentation and comparison with the source data used to create the phantom.

**Results:**

Realistic relative HU values were measured although a subtraction of 60 was required to obtain equivalence with the expected values (gray matter 32.9–35.8 phantom, 29.9–34.2 literature). Segmentation of images acquired at different kVps/mAs showed excellent agreement with the source data (Dice Similarity Coefficient 0.79 for gray matter). The performance of two scanners with two segmentation methods was compared, with the scanners found to have similar performance and with one segmentation method clearly superior to the other.

**Conclusion:**

The ability to use 3D printing to create a realistic (in terms of geometry and attenuation properties) head phantom has been demonstrated and used in an initial assessment of CT segmentation accuracy using freely available software developed for MRI.

## 1 introduction

X‐ray computed tomography (CT) and magnetic resonance imaging (MRI) are commonly used imaging techniques for the assessment of pathological brain disorders. Modern CT has many advantages over MRI, including lower cost of both the imaging system and patient examination, fewer motion artifacts due to faster acquisition times (up to two orders of magnitude), greater availability, higher spatial resolution, and fewer limitations related to claustrophobia and the presence of ferromagnetic materials in the body. The disadvantages of CT include lower contrast/noise and the exposure of the patient to ionizing radiation. However, while radiation exposure is a concern, the risk/benefit ratio is age and organ dependent, usually justifying the use of CT to study brain pathology in the elderly.^1^, ^2^

Structural neuroimaging is recommended as part of the clinical evaluation in all patients with suspected dementia in the United Kingdom.^3^ Currently, unenhanced CT is used as the first line of neuroimaging in dementia and an estimated 150 000 scans are carried out per year in the United Kingdom.^4^, ^5^ Images are generally inspected visually, and reports framed in terms of the location of substantial atrophy, tissue abnormality, or ventricular enlargement. The number of imaging studies carried to investigate neurodegenerative disease, particularly for dementia, looks certain to expand considerably due to an increased incidence^6^ while the number of radiology vacancies continue to grow.^7^

Over the past two decades, the development of several automated techniques for the analysis of structural MRI data has led to a proliferation of studies on the neuroanatomical basis of both neurological and psychiatric disorders.^8^, ^9^ The most widely used technique is voxel‐based morphometry (VBM) which involves a voxel‐wise comparison of the local volume or concentration of gray and white matter (GM and WM) between groups of subjects.^10^, ^11^ Voxel‐based morphometry has been used successfully to investigate a wide number of disorders such as Alzheimer's disease (AD),^12^ Parkinson's disease,^13^ depression,^14^ and multiple sclerosis.^15^

Clinical application of these methods would appear promising in the assessment of neurodegenerative conditions; automated brain morphometry is increasingly recognized as a biomarker for AD^16^ and atrophy measurement from serial scans is an attractive way to characterize diseases such as multiple sclerosis.^17^, ^18^ However, while a number of these sophisticated methods of analysis are available to quantify local and global atrophy from MRI, relatively little progress has been made to integrate these into clinical workflows due to special hardware requirements, prohibitively long processing times and dependency on specific acquisition techniques.^19^ The accuracy and precision of these methods have not been well characterized.^20^ Software approved for clinical use has been shown to lead to errors in the evaluation of neurodegenerative conditions.^21^

Validation of MRI segmentation is difficult as the ground truth (the precise nature of the underlying anatomy) is not known although reliability and reproducibility of the FMRIB Software Library (FSL), statistical parametric mapping (SPM), and FreeSurfer have been benchmarked using human subjects.^22^ Although digital phantoms are widespread for MRI,^20^, ^23^, ^24^ they do not test the actual imaging chain as the scanner itself is simulated. Due to the difficulties in both creating and handling materials with realistic MR properties, no realistic full brain physical MRI phantoms are available.

An alternative would be to apply automated segmentation and volumetry to x‐ray computed tomography of the brain, an approach that has been proposed but has yet to be clinically implemented for single subjects.^25^, ^26^ The accuracy of CT segmentation will depend, to some extent, on the ability of CT images to accurately depict the structures of the head. This in turn will depend on the scanner used and the exposure and reconstruction factors selected. The delineation of soft tissue structures will depend on material contrast, edge resolution, and image noise, which are in turn affected by the peak tube potential (kVp), filtration, tube current (mA), rotation time, reconstructed slice width, and the reconstruction algorithm, including iterative methods and any other postacquisition image processing. Furthermore, any segmentation may depend on the orientation of the patient in the scanner and any beam hardening or movement artefacts.

Segmentation approaches based on open source software such as statistical parametric mapping (SPM) have been applied to CT. The availability of CT‐specific templates, registration, and rewindowing functions in the SPM clinical toolbox^27^ is an important step forward and has led to the further creation of procedures for the automated delineation of stroke and atrophy.^28^ Another recent study has used FMRIB's automated segmentation tool (FSL FAST) to segment CT scans without the use of prior maps, although the results appear somewhat disappointing.^29^ A landmark study uses the standard segmentation procedure in SPM8 to successfully delineate GM, WM, and CSF and then demonstrates the superiority of CT‐VBM compared to MRI‐VBM for a group study of AD patients compared to controls.^26^ However, to segment the images various preprocessing/resampling steps were required and the SPM settings had to be varied for individual subjects. This makes the analysis both time consuming and potentially inaccurate at the individual level. The study authors have recently revised their method to use the default SPM12 settings to segment CT scans to increase the accuracy of the standardization of DaTscan imaging, but have not repeated their work with dementia scans.^30^ Another study uses a segmentation protocol based on SPM12 combined with topologically constrained tissue boundary refinement to delineate WM, GM, and cerebrospinal fluid (CSF) and directly assesses accuracy by comparing CT segmentation for MRI segmentation in the same subjects.^31^ Before clinical use can be contemplated a method is required to test the accuracy and reproducibility of the entire imaging chain, including the scanner, reconstruction, and the software for segmentation and quantitative analysis of the brain.

The production of realistic medical models by 3D printing (3DP) has expanded greatly in recent years; however, this concentrates almost exclusively on visualization for surgical planning and creation of custom tailored implants and prostheses.^32^ Several studies describe the creation of anthropomorphic and even patient‐specific phantoms for use in either CT or radiotherapy dosimetry. The limitation of the phantoms presented in these studies is that they do not allow for complex nested structures with multiple material properties, as would be required to simulate the brain. The earliest of these uses a fused deposition modeling printer to recreate the RANDO phantom in a two stage process where 3DP was used to produce the exterior surfaces from acrylonitrile butadiene (ABS) which were used as a mould.^33^ The ABS phantom was verified as soft tissue equivalent by comparing dose measurements using both the original RANDO phantom and the printed/moulded phantom.

Another study used a real human skull as a basis for a mould for the construction of an anthropomorphic head phantom using a mixture of dolomite and polymethyl methacrylate.^34^ A 1:1 mixture resulted in the creation of a phantom with realistic attenuation coefficients. Two 3DP technologies — digital light processing (DLP) and Polyjet — were used to print patient‐specific models of the spine which were then embedded in an acrylic body phantom.^35^ Realistic Hounsfield unit (HU) measurements were achieved, with DLP producing higher values typical for younger patients and PolyJet producing the lower values characteristic of older patients.

A patient‐specific radiotherapy phantom, consisting of 11 3DP sagittal slices through the chest and neck, was created using segmented DICOM data and printed using polylactic acid (PLA).^36^ This produced an accurate single tissue phantom with relatively inaccurate HU simulation; the typical deviation was 120 HU. A similar single tissue patient‐specific phantom of the head and neck was printed in two parts using the proprietary ABSplus material.^37^ Hounsfield unit discrepancies were also high at approximately −300 HU. Finally, a commercial anthropomorphic head phantom containing the bone and hollow of the maxillary sinus was reproduced as a single tissue 3DP phantom with slots for dosemeters fabricated using PLA.^38^ The HU discrepancies between the commercial and 3DP phantoms approached 2000 HU in bone.

Of more interest is a recent study using 3D printing techniques, that created a life‐size phantom based on a clinical CT scan of the thorax of a patient with lung cancer.^39^ This was assembled from bony structures printed in gypsum, lung structures consisting of airways, blood vessels >1mm, an outer lung surface, three lung tumours printed in nylon, and soft tissues represented by silicone (poured into a 3D‐printed mould). Correspondence between the source image and phantom HU was good across the range −478 to +730.

Another study used a Polyjet printer to produce tissue equivalent phantoms representing the anatomical texture present in the lungs and soft tissue.^40^ Hounsfield unit measurements were taken for a range of blended materials. The available blended proportions were discrete rather than allowing fine tuning of materials. This showed HU in the range 15–95 are possible, depending on the kVp of the CT scanner. It has been demonstrated that Polyjet printing is also able to simulate bone, but it is not possible to print this at the same time as the soft tissue structures.^35^ However, Polyjet printing offers a way of 3D printing a multiple material phantom with structures close in x‐ray attenuation to those of the brain and CSF.

This work aims to use 3D printing to create a realistic anthropomorphic phantom representing the CT properties of a normal human brain and skull. Properly developed, this type of phantom will allow the optimization and validation of CT segmentation across different scanners and disease states. Although the lack of contrast available from CT means that procedures such as hippocampal segmentation and cortical thickness analyses may never be possible, segmentation will allow the use of disease‐specific atlases^41^ and approaches based on sulcal morphology.^42^ If sufficient realism can be attained with the phantom, imaging the resulting phantom on different scanners and using different acquisition parameters will enable the validation of the entire processing chain in the proposed clinical implementation of CT‐VBM.

## 2 MATERIALS AND METHODS

### 2.A Materials and 3D printing

All 3D printing was carried out on a Stratasys Objet printer (Stratasys Ltd, Israel) by the Bristol Robotics Laboratory (Bristol, UK) using Tango+ and VeroWhite materials. This printer has a maximum resolution of 0.1 mm. Initially, six material blends were chosen based on a recent study^40^ and printed as single material cylinders of diameter 5cm. These were scanned on a Siemens Somatom AS + CT scanner (Siemens, Germany) at a range of kVp values. For each cylinder, Image J was used to measure the mean HU and standard deviation in a circular ROI for a stack of 15 × 3 mm images along the cylinder. The mean values along the stack were calculated and are shown in Table I.

**Table I:** Hounsfield units of blended materials at different kVp values.

Material	9740		9750		9760		9770		9785		9795	
kVp	Mean	StdDev	Mean	StdDev	Mean	StdDev	Mean	StdDev	Mean	StdDev	Mean	StdDev
70	52.4	5.0	54.1	5.2	55.7	5.2	61.9	5.5	63.5	5.6	71.6	5.5
80	63.4	4.1	65.3	4.3	67.2	4.3	72.4	4.4	74.6	4.4	82.4	4.4
100	75.3	3.0	77.4	3.2	79.6	3.3	84.8	3.3	87.5	3.3	94.9	3.2
120	81.9	2.4	84.2	2.7	86.4	2.7	91.6	2.7	94.8	2.8	101.9	2.7
140	86.1	2.2	88.4	2.5	90.7	2.5	96.0	2.3	99.4	2.6	106.4	2.4

The materials 9740, 9770, and 9795 were chosen to present CSF, WM, and GM, respectively. Depending on the kVp used, a simple subtraction of 50–60 HU from the scanned images of the phantom should yield corrected HU values approximately 10 HU apart across the required range. The measured HU was broadly in agreement with the literature (see Table III) and shows both a progression with the proportions of the blend, and an increase with kVp. No material accurately represents the HU of GM, WM, or CSF, but the difference in HU between each, at 10–15 HU can be readily simulated. It should be noted that the measured HU values in this study are consistently around 30 higher than the values in the recent study.^40^ This may be due to different scanner manufacturers (Siemens vs General Electric), different sample geometries leading to different scatter fractions, the use of different model Stratasys printers and a 4 yr gap between studies during which time the printed material composition may have been altered.

### 2.B Segmentation using SPM12

Statistical parametric mapping segmentation uses a probabilistic framework combining image registration, tissue classification, and bias correction within the same model. Voxel intensity values are used to assign their probabilities of belonging to one of several tissue classes via estimation of the parameters of the intensity distributions of each class. An objective function is derived from a mixture of Gaussian random variable models, a parameter optimization process is then used to minimize the value of this function. A set of *a priori* tissue probability maps specified in a standard space is used to assist the classification. Statistical parametric mapping uses standard brains from the Montreal Neurological Institute (MNI). The MNI defined a new standard space by using a large series of MRI scans on normal controls. The objective function assists this process by weighting the probability maps of MNI space according to Bayesian inference principles and then deforming them so that they match the volumes being segmented.^10^, ^31^ For this work two sets of *a priori* tissue probability maps were employed; first, the default maps provided with the SPM12 software were used. Second, compatible maps from a study that created a database of adult, age‐specific MRI brain, and head templates were used.^43^ The study participants included healthy adults from 20 through 89 yr of age. The templates were done in 5‐, 10‐yr, and multi‐year intervals from 20 through 89 yr, and consist of publicly available average T1W images for the head and brain, and *a priori* tissue probability maps. The maps from the oldest age group (80–89 yr) were used.

### 2.C Alzheimer’s disease neuroimaging initiative (ADNI) control T1 MRI data

Magnetic resonance imaging scans of 10 control subjects between the ages of 56 and 68 were selected and segmented using default SPM12 parameters. Bias correction was used with light regularization and 60 mm FWHM cutoff. The tissue probability maps provided by SPM were used depicting GM, WM, CSF, bone, soft tissue, and air/background which used 1, 1, 2, 3, 4, and 2 Gaussians per class, respectively. Where more than one Gaussian is specified this means the tissue probability map may be shared by several clusters. Markov random field cleanup was enabled and the brain extraction cleanup was disabled. Warping regularization parameters were set to the default values, affine regularization was to European brain using the ICBM space template and the sampling distance was three. Further details can be obtained from the SPM12 manual supplied with the software.^44^

Segmentation produced GM, WM, and CSF segments in standard space with tissue densities between 0 and 1. These segments were averaged and the normalized mean square error (NMSE) between the individual segments and the average were calculated. The individual T1 scan with the lowest total NMSE for the three tissue types was selected to be the basis of the phantom. This scan had in‐plane resolution of 1.02 × 1.02 mm and a 1.2 mm slice thickness. NMSE, where s_i_ and m_i_ are the ith voxels of the individual segmented and mean images respectively, is defined below.

$$NMSE=\frac{\sum_{i}{\left(m_{i}-s_{i}\right)}^{2}}{\sum_{i}m_{i}^{2}}$$

### 2.D Creation of print files from segments

To avoid overlapping maps, after the segmented dataset was selected, each segment was thresholded at various values between 0.1 and 0.6 using the SPM12 imcalc function. The imcalc function was then used to check for zero value voxels in the brain; any zero values would be printed with support material, the attenuation properties of which are unknown. After visual assessment of the resulting maps 0.4 was chosen as the threshold, that is, values below 0.4 were set to zero and values above were set to 1. The grayscale modelmaker function in 3d slicer was then used to create stereolithography (STL) files for each tissue type using a threshold of 1, 15 smoothing iterations, and a decimation fraction of 0.25.^45^ Print files for each tissue type were then created from the STL files.

### 2.E 3DP process

**Fig. 1:**
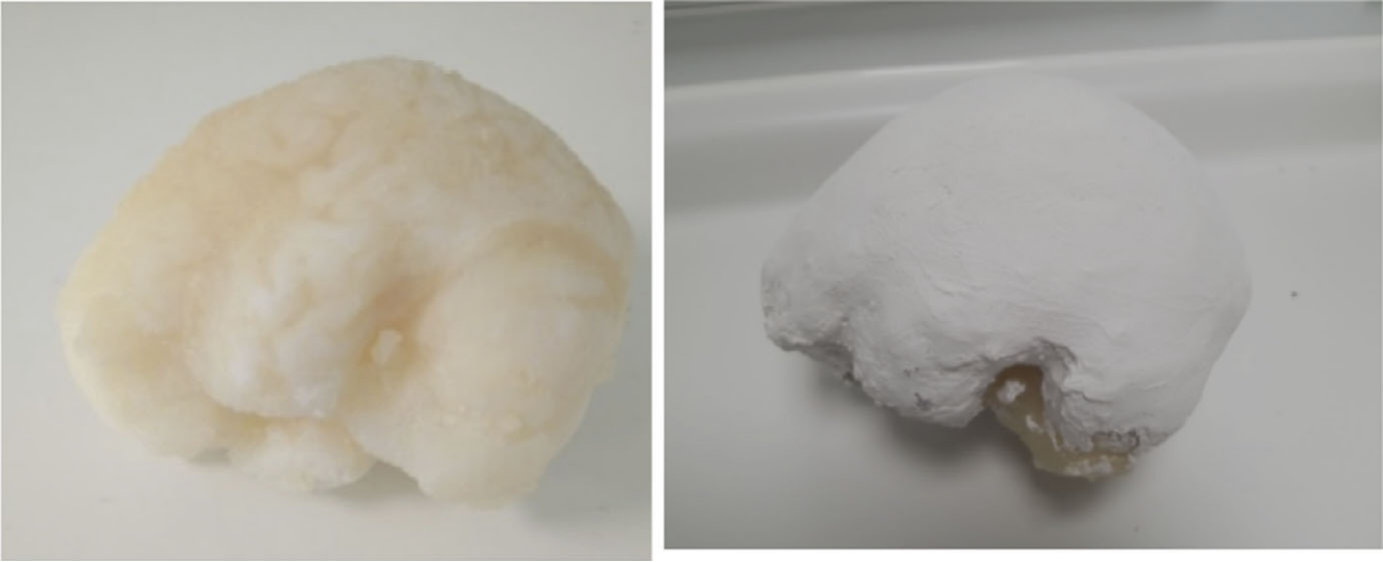
Three‐dimensional printing brain (left) and the completed phantom after coating with plaster of Paris (right). [Color figure can be viewed at wileyonlinelibrary.com]

The phantom was printed and the support material removed. However, a simulated skull was required to reproduce the attenuation, beam hardening, and scatter in clinical scans of the head. Plaster of Paris offered a simple method to create a skull around the 3D printed brain; the HU of this material had previously been shown to be similar to that of the skull.^46^ Layers of plaster of Paris impregnated bandages (Gypsona, France) were applied to the phantom to create a skull of approximately 3 mm thickness. The base of the brain was not covered. The phantom is shown as Figure 1.

### 2.F CT scanning

The assembled phantom was imaged using a Toshiba Aquilion One CT scanner using a 0.5 mm slice width and 0.468 mm in‐plane resolution (scanner 1). Subsequently the phantom was imaged using a General Electric Discovery 670 SPECT/CT system using a 0.625 mm slice width and 0.625 mm in‐plane resolution (scanner 2). For both scanners, the phantom was placed in the patient head rest and oriented to represent normal patient positioning, that is, supine without head tilt. The scan time was 1 s and the kVp and mA were both varied and matched as far as possible between the two scanners (see Table II). For scanner 1, reconstruction was carried out using the brain kernel. For scanner 2 only 1 kernel (“standard”) was available. Additional scanning to study the kV variation of the phantom materials was carried out using a Siemens Symbia SPECT/CT as the other scanners were not available. As for scanner 2, only one kernel was available.

**Table II:** Acquisition parameters for the two computed tomography scanners used to scan the phantom.

Acquisition ID	kVp — scanner 1	mA — scanner 1	kVp — scanner 2	mA — scanner 2
A	100	300	100	255
B	120	150	120	150
C	120	300	120	300
D	135	300	140	280

### 2.G Image processing

SPM12 was used to convert DICOM files exported from the scanner to analyze format. The imcalc function was then used to subtract 60 HU globally from each scan. The SPM clinical toolbox was then used to permanently transform image intensity values from HU by changing the values in the image file.^27^, ^47^ This is equivalent to a radiologist choosing brightness and contrast levels to emphasize tissue contrast, a process usually referred to as windowing^48^ or rewindowing. The transformation increases the dynamic range of the range of HU that are of interest. Three factors are used in this operation; dark units, mid units, and a scaling factor with defaults of 1000, 100, and 10, respectively. The dark unit value is subtracted from the image and the dynamic range of the HU values in the mid unit range is increased using the scaling factor. Further details can be found in the code files of the clinical toolbox, accessible from the SPM website.^44^

The images before and after permanent rewindowing were then segmented twice; first using the default SPM12 settings and second using age‐specific prior tissue maps.^43^ This resulted in four segmentations for each scan — rewindowed default (RWD), rewindowed age‐specific maps (RWX), no rewindowing default (D), and no rewindowing age‐specific maps (X). As the scanned images were not in register with the phantom source data, the SPM12 coregistration function was used to register an averaged GM map from the phantom acquisitions to the source data GM map using rigid body affine‐only transformations. The derived parameters were applied to all segments and unsegmented scans in order to coregister them to the source data. The segments derived from the scanned images were binarized (with intensities >0.4 set to 1) were compared to the source data used to create the phantom print files in order to calculate the DSC.

In order to visualize the accuracy of the segmentation of individual brain structures, regions of interest from the Automatic Anatomic Labelling toolbox^49^ were inverted from standard space into patient space using the deformation matrices generated during the initial SPM12 segmentation of the source MRI and then with the deformation models obtained from CT segmentation. After inversion the ROIs were converted to surface models using the same process as described in Section 2.D. The surface models derived from the two segmentations were then compared visually using 3d slicer.

### 2.H Dice similarity coefficient (DSC)

The difference between various single segmented images and the source data used to create the phantom was characterized by a single number, the DSC.^50^ The DSC measures the spatial overlap between two segmentations, A and B, and is defined as DSC(A,B) = 2(A ∩ B)/(A + B), where ∩is the intersection.^51^

## 3 RESULTS

**Fig. 2:**
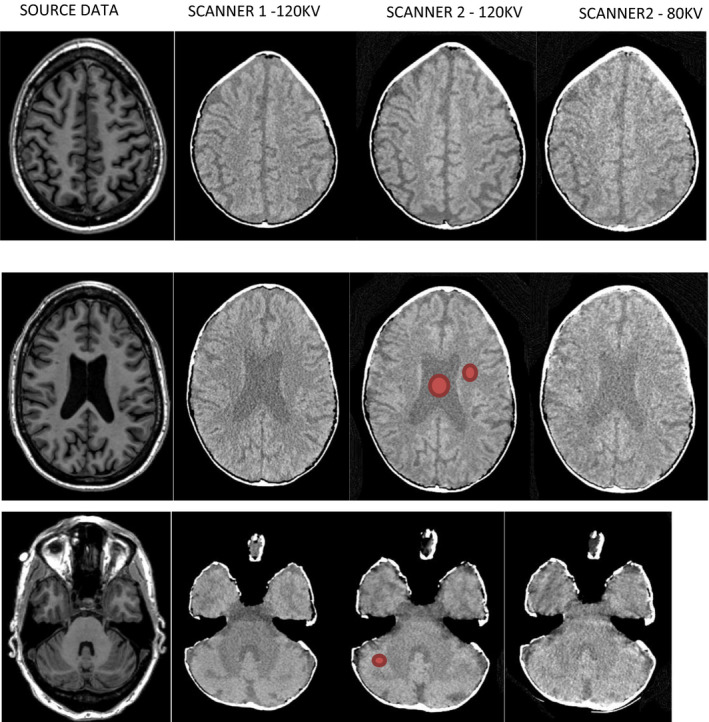
Comparison of the source magnetic resonance imaging (column 1) and phantom scan C (120 kV, 300 mAs) for scanner 1 (column 2) and scanner 2 (column 3) with an 80 kV acquisition on scanner 2 (column). The three rows depict different slices at different levels in the head/phantom. As the printer was only capable of printing three different types of plastic no nonbrain structures —– such as the eyes or skull —– were printed. Computed tomography scans have 60 HU subtraction and are displayed with a window level of 30 HU, window width 90 HU. Representative ROIs used for determination of the mean HU for each tissue type are shown in red. [Color figure can be viewed at wileyonlinelibrary.com]

Figure 2 shows a comparison of the source MRI and CT scans of the phantom in the two scanners. The CT scans appear to be an accurate reflection of the source data with faithful reproduction of tissues and anatomy. There appears to be a gap between the plaster of Paris and printed CSF in some areas. This may be air or support material that has filled undetected gaps between the STL files used to create the phantom.

Table III compares the range and mean of measured HU in the phantom for acquisition ID C for both scanners with simulated values.^52^ Additional data are taken from published studies of patient data; scanner A was a Phillips Brilliance 64‐channel CT scanner at 120 kV and the maximum obtainable tube current,^53^ scanner B was a GE Lightspeed VCT and Scanner C a GE Discovery CT750 HD both operating at 120 kV.^54^ Additional values for the skull^52^, ^55^ and CSF^52^ were also obtained.

**Table III:** Comparison of expected and measured HU values for scan C for both scanners (right hand columns). The expected values for scanners A to C are from patient studies. Simulated values are from Monte Carlo simulations of clinical dose distributions. The measured phantom values for scanner 1 and scanner 2 are after a subtraction of 60 HU.

Tissue	Published data				This study	
	Mean CT number scanner A^53^ (120 kVp)	Mean CT number scanner B^54^ (120 kVp)	Mean CT number scanner C^54^ (120 kVp)	Simulated value (120 kVp^52^, not specified^55^)	Mean scanner 1 (120 kVp)	Mean scanner 2 (120 kVp)
Gray matter	29.9 ± 3.8	33.2 ± 0.74	34.18 ± 0.97	40^52^	35.8 ± 9.9	32.9 ± 6.7
White matter	22.7 ± 3.8	25.06 ± 0.6	26.11 ± 0.93	34^52^	29.3 ± 9.5	27.4 ± 6.1
Cerebrospinal fluid	—	—	—	13^52^	22 ± 10.4	19.5 ± 6.9
Cancellous bone		—	—	568–828^55^, 999^52^	690	715

**Fig. 3:**
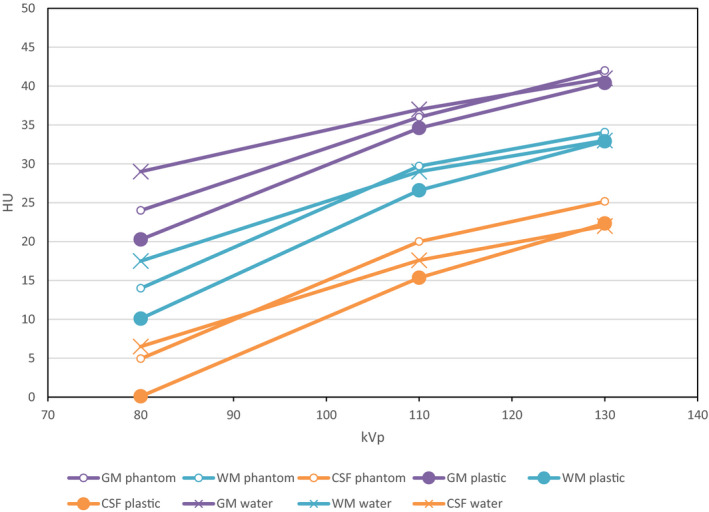
Variation of measured HU for the phantom and for the plastic samples selected for each tissue type — data acquired on a Siemens Symbia SPECT/CT. The phantom was scanned in air (these results are labeled “phantom”) and subsequently the materials were scanned in a 20 cm water tank (these results are labeled “water”). Results are after a subtraction of 60 HU. [Color figure can be viewed at wileyonlinelibrary.com]

After an adjustment of 60 HU reasonable agreement is seen and the images appear realistic. Figure 3 shows the variation of HU values with kV for the phantom and plastic source material (scanned in air) and plastic source samples (scanned in a 20 cm diameter water phantom). This was carried out using a Siemens Symbia SPECT/CT system. Good agreement between the plastic sample measurements at 80kV with the initial material assessment in Table I which also used a Siemens scanner. The kV variation is as expected from a previous study^40^ with the gray/white ratio diminishing with increasing kV. The printed and water phantom values are higher than initially measured material values particularly at 80 kV, which may reflect beam hardening in the phantoms.

**Fig. 4:**
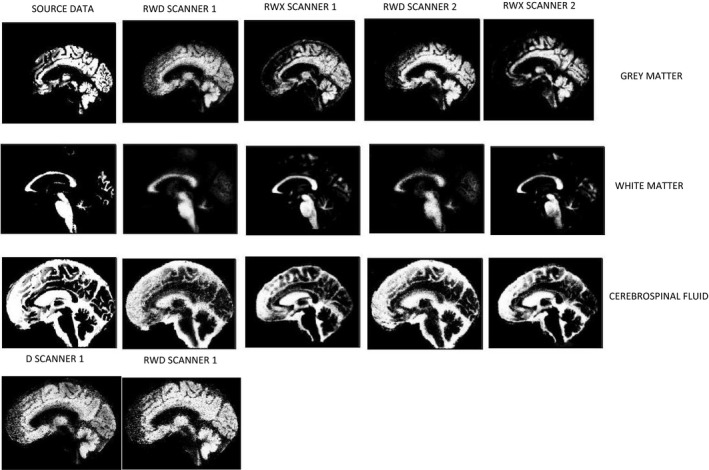
Comparison of tissue maps in patient space for the source magnetic resonance imaging (left) and after segmentation of acquisition C using the rewindowed default (RWD) method for scanner 1, RWD for scanner 2 then the rewindowed age‐specific maps (RWX) methods for each scanner. Top row is gray matter, second row is white matter, and third row is cerebrospinal fluid. It can be seen that the RWX segmentations are noticeably less blurred. The bottom row compares default segmentations with (RWD) and without rewindowing (D) for scanner 1, showing greater uniformity particularly around the motor/sensory cortex for RWD.

**Fig. 5:**
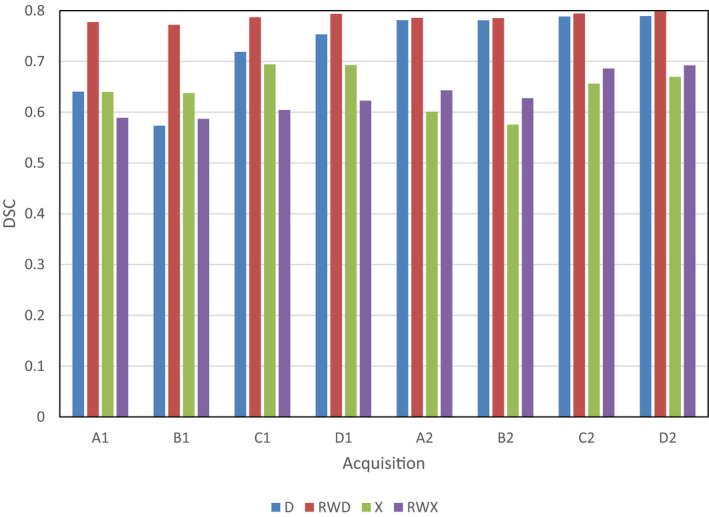
Variation of dice similarity coefficient (DSC) results for GM segments. Different acquisition settings are labeled A to D for scanners 1 and 2. Segmentations settings are default (D), default with rewindowing (RWD), age‐specific maps (X) and age‐specific maps with rewindowing (RWX). [Color figure can be viewed at wileyonlinelibrary.com]

**Fig. 6:**
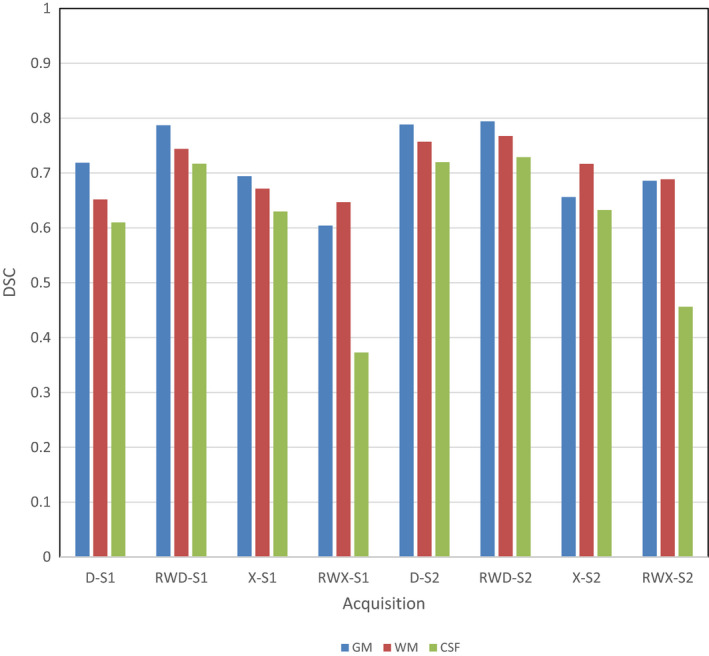
Dice similarity coefficient results for acquisition C for all scanners, tissue types, and segmentation methods. Scanners are referred to as S1 and S2. Segmentations settings are default (D), default with rewindowing (RWD), age‐specific maps (X) and age‐specific maps with rewindowing (RWX). [Color figure can be viewed at wileyonlinelibrary.com]

Figure 4 shows a comparison of the source MRI tissue maps and the maps obtained after scanning the phantom on both scanners and segmenting using the RWD and RWX settings on both scanners; they are from acquisition C as the kV and mAs are the same for both scanners and are typical of clinical practice.^1^ Visual assessment of these images shows generally good agreement. The RWD segmentations show greater blurring between GM and WM than the RWX segmentations, but with the RWX segmentations showing gross differences at the edge of the brain. These observations are reflected in Fig. 5, where the highest DSC values for the GM segmentations result from the RWD method for both scanners, with a peak DSC of around 0.8. Figure 6 shows the DSC results for acquisition C in more detail for each segmentation type. As would be expected from Fig. 4, the DSC for the RWX method is around 0.4 for both scanners for the CSF, reflecting inaccurate segmentation at the edge of the brain for the age‐specific maps.

**Fig. 7:**
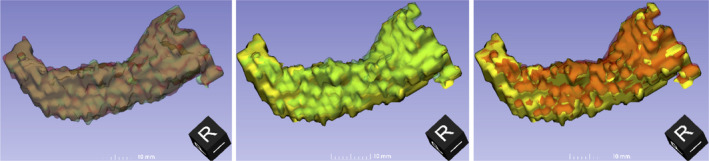
Comparison of the segmentation of the left hippocampus from the source magnetic resonance imaging (MRI) used for printing (solid yellow) with the segmentation using rewindowing and default settings (RWD) of acquisition C for scanner 1 (translucent green) and scanner 2 (translucent red). The orientation of each three‐dimensional view is shown by the black cube. [Color figure can be viewed at wileyonlinelibrary.com]

Finally, the accuracy of the segmentation of individual brain structures is illustrated in Fig. 7. This shows a 3D overlay of the left hippocampus from the source data and from the C acquisitions for both scanners. Visually, the overall shape of the segmentations appears similar but both CT segmentations appears offset from the source data by approx. 1 mm along the hippocampal axis and 2 mm laterally. This is comparable to the voxel size of the segmentations (1.5 mm) used to create the phantom itself and to assess CT scans of the phantom, and considerably greater than the maximum print resolution of 0.1 mm. The scanned segmentation does not appear to show any substantial distortion relative to the source data.

## 4 DISCUSSION

This study has used two scanners with consistent acquisition parameters and two segmentation methods. Method 1 used the default SPM12 segmentation and method 2 replaced the tissue prior maps with age‐specific alternatives. These maps were used after a number of mis‐segmentations of CSF for patients with large ventricles were observed. For the mis‐segmentations areas of ventricular CSF toward the edge of the brain (i.e., outside the areas in the SPM12 CSF prior map) were mis‐classified as WM. The age‐specific maps had larger ventricles and resulted in visually more accurate segmentations of ventricular CSF.

The measured DSC in this study approach 0.8 for GM segmentation for rewindowed scans. These are lower than values obtained in a recent study comparing an enhanced segmentation approach based on SPM12 but incorporating neuroanatomy‐constrained correction of tissue boundaries based on the local topological properties of the GM/WM interface.^31^ Their approach obtains higher DSC for WM and CSF (0.85 and 0.91, respectively), indicating the success of their method, with a similar DSC measured for GM of 0.87. It is worth noting, however, that in order to demonstrate the utility of their method the study authors had to collect data from consented patients and had to exclude poor quality scans.

The highest DSC values — between 0.7 and 0.8 for all tissues — were calculated for scanner 2 using rewindowing and default segmentation. These values were only slightly higher than those calculated for scanner 1 and would be unlikely to cause a discernible difference between analyses carried out on segmented data from the scanners. It is interesting to note that a SPECT/CT system has comparable performance to a dedicated CT scanner and could lead to the routine acquisition of diagnostic CT data from SPECT/CT systems at the same hospital visit for HMPAO SPECT scanning for dementia patients.

As previously noted, in Fig. 4 the peripheral CSF in the source data is represented in the RWD results but not in the final two columns which illustrate inaccurate CSF segmentation using the RWX methods. This is reflected in the DSC values in Fig. 6, with values as low as 0.38 for scanner 1. This effect can sometimes be seen when segmenting MRI scans using the old age priors as opposed to the default SPM12 maps and represents an advantage to using the relatively blurred SPM12 maps for normal subjects. It may be that the optimal approach would be to blur the old age maps, or create new maps with relatively large ventricles by averaging the results of successful segmentations of older subjects.

It appears that rewindowing improves contrast (or at least makes the images have a similar dynamic range to MRI) and uniformly improves DSC results. Additionally, the DSC results from scanner 2 are higher for some settings, possibly reflecting the larger voxel size and lower noise.

The representation of the skull is relatively crude, as it was simply moulded around the 3D printed brain and CSF. Although the plaster of Paris impregnated bandage was applied directly to the printed phantom, and manually moulded to its contours, the scans show an air gap between the skull and CSF. This is due to contraction either of the printed material or, more likely, the plaster of Paris. In addition, it is thinner than a typical skull although more plaster of Paris could easily be added. However, while these shortcomings reduce the realism, they are unlikely affect the accuracy of GM and WM segmentation and will still provide the required attenuation. Although further work is required to fine tune the scanned HU values of the three main tissues and to 3D print simulated bone, we were able to create the phantom for approximately £900 using commercial 3D printing equipment, open source software, and freely available imaging data. It took 2 hours to create the STL files from the downloaded ADNI data, although this procedure could be automated.

As outlined in the introduction, although the contrast available from MRI is considerably greater than CT, subtle differences in image acquisition and even scanning the same individual but on different scanners has the potential to yield different results.^56^ Physical phantoms that can adequately simulate the human brain are not available, although digital brain phantoms have been used to demonstrate that SPM, FSL, and FreeSurfer underestimate GM and overestimate WM volumes with increasing noise.^24^ Results from simulated images are always limited in their application because simulated images cannot capture the full complexity of real MR images and it has been demonstrated that, in the case of FSL FAST, it is not sufficient to use simulated images to get an idea of how a segmentation algorithm will perform on real datasets.^20^ A study of the effects of deviations from ideal image quality on the output of the brain boundary shift integral method showed that measurement errors can exceed the disease effect in AD.^17^ The ADNI MRI protocols^57^ demonstrate that standardization can be successfully applied in this area but may be too stringent for routine clinical use.

It may well be possible to use phantoms to measure parameters that could be used as exclusion criteria in the clinical use of CT analyses, thereby increasing sensitivity, specificity, and clinical confidence. It appears from this work that rewindowing is a necessary prerequisite for the use of CT segmentation and that DSC levels of 0.8 are achievable and could serve as an initial threshold.

The effects of neuroimaging on clinical confidence analyses are not an area that has been investigated rigourously, the effects of analyses even less so.^58^, ^59^, ^60^, ^61^ The literature appears to concentrate more on novel methods rather than demonstrating the usefulness of existing ones.

It would be relatively straightforward to create multiple phantoms of the same subject with progressive atrophy; the atrophy could be simulated from a “base” scan or by the assessment of multiple patient scans from the ADNI database. A recent publication has indicated that the changes in brain structure in Alzheimer's patients can be detected in periods as short as 6 months on serial MRI scanning^62^ and the comparison of serial CTs also show promise.^63^ Care would be required when creating multiple STLs/phantoms with only small differences as inaccuracies in the preprocessing may mask these changes.^64^ It may be the case that in the future the systematic use of phantoms to minimize the variance of image/segmentation quality across scanners may improve the accuracy of patient analyses using approaches such as deep learning.^65^, ^66^

A recent study on CT segmentation indicates that higher accuracy than achieved in this study may be obtained via enhancements to SPM12 or the development of a machine learning approach utilizing a support vector machine.^67^ These studies used data pooled across multiple scanners and have limited applicability as the effects of different scanners, and acquisition protocols cannot be examined without further data collection from human subjects. More importantly they utilize a ground truth for DSC calculation for CT segmentation based on MRI segmentation for the same patient. These datasets are time consuming and expensive to collect and represent arguably a less reliable ground truth than the CT phantom described in this work.

## 5 CONCLUSIONS

We have demonstrated that 3D printing can be used to create a highly realistic — in terms of both physical characteristics (HU) and anatomical accuracy — physical CT phantom of the human brain. As the precise internal structure of the phantom is known it was possible to demonstrate the similarity of the two scanners and the improved performance of one of the segmentation methods compared to the other. To our knowledge this is the first study of these areas.

This phantom has immediate applications in dose vs image quality optimization for visual image interpretation and the selection of minimum acceptable segmentation accuracy for existing and proposed clinical CT segmentation workflows.
